# Parathyroid Adenoma Detected in ^68^Ga-PSMA PET/CT but Not in the Dedicated Imaging Modalities

**DOI:** 10.3390/diagnostics14151690

**Published:** 2024-08-05

**Authors:** Maja Cieślewicz, Natalia Andryszak, Kacper Pełka, Ewelina Szczepanek-Parulska, Marek Ruchała, Jolanta Kunikowska, Rafał Czepczyński

**Affiliations:** 1 Department of Endocrinology, Metabolism and Internal Diseases, Poznan University of Medical Sciences, 61-701 Poznan, Polandmruchala@ump.edu.pl (M.R.);; 2 Department of Nuclear Medicine, Affidea Poznan, 61-485 Poznan, Poland; 3 Laboratory of Centre for Preclinical Research, Department of Research Methodology, Medical University of Warsaw, 02-091 Warsaw, Poland; 4 Department of Nuclear Medicine, Medical University of Warsaw, 02-091 Warsaw, Poland

**Keywords:** parathyroid adenoma, hyperparathyroidism, PET/CT, PSMA

## Abstract

Background: Primary hyperparathyroidism is a common endocrine disorder characterised by excessive parathormone secretion that results in hypercalcemia, primarily caused by parathyroid adenoma. Accurate localisation of hyperfunctioning tissue is essential for curative surgical treatment. Although conventional imaging modalities like ultrasonography and ^99m^Tc-MIBI scintigraphy (SPECT) along with ^18^F-fluorocholine PET/CT are commonly employed, there are cases with false-negative imaging results. Case presentation: This case report presents a patient with primary hyperparathyroidism and a parathyroid adenoma detected solely through ^68^Ga-PSMA-11 PET/CT, typically used for prostate cancer diagnosis. The lesion observed in the PET/CT was confirmed as a parathyroid adenoma through laboratory evaluation, while other imaging techniques failed to detect it. Conclusions: This finding suggests that the PSMA ligands’ particular affinity for neovascularisation in focal changes may facilitate the visualisation of parathyroid adenomas. The utilisation of ^68^Ga-PSMA-11 PET/CT in primary hyperparathyroidism could potentially improve the preoperative localization of parathyroid adenomas when conventional imaging methods are inconclusive.

## 1. Introduction

Primary hyperparathyroidism (PHPT) is defined as excessive parathormone secretion, causing hypercalcemia. Clinically, it may demonstrate a vast range of symptoms, and it occurs primarily due to parathyroid adenoma and rarely as a consequence of parathyroid hypertrophy or cancer. Surgery remains the only curative treatment; therefore, precise parathyroid adenoma localisation is crucial [1,2].

The vast majority of patients with PHPT are asymptomatic because most patients are incidentally discovered when routine laboratory work reveals hypercalcemia. However, chronic hypercalcemia may cause multi-system disorders involving skeletal, renal, gastrointestinal, neurological, and psychiatric manifestations. The most common clinical demonstrations are skeletal and renal. Osteoporosis and osteopenia increase the risk of pathological fractures, especially clinically silent vertebral fractures. The main renal manifestations of PHPT are hypercalciuria and nephrolithiasis. The neuropsychological system may be affected by hypercalcemia, causing muscle weakness and atrophy, but also depression, anxiety, fatigue, or sleep disturbance [1,2].

Parathyroidectomy remains the only cure for PHPT and is recommended in all symptomatic patients. Imaging modalities used to localise the parathyroid abnormalities include ultrasonography and ^99m^Tc-MIBI scintigraphy (SPECT). Recently, ^18^F-fluorocholine PET/CT has been introduced as an even more effective technique [3,4,5]. ^18^F-choline PET/CT presented better diagnostic accuracy than neck ultrasound and parathyroid scintigraphy. The method showed excellent sensitivity and positive predictive value, including patients with nodular goitre, chronic thyroiditis, and prior unsuccessful parathyroidectomy [3,4]. However, in clinical practice, there are still some patients presenting with laboratory evidence and symptoms of primary hyperparathyroidism and still false-negative imaging.

Glutamate carboxypeptidase II is a transmembrane glycoprotein primarily recognised for its overexpression in prostate cancer cells that obtained here another name: prostate-specific membrane antigen (PSMA). While PSMA was studied in the context of prostate cancer, glutamate carboxypeptidase II (GCPII) was researched in brain and neurological diseases. Researchers invested significant time in establishing that PSMA and GCPII are encoded by the same gene and represent the same protein, responsible for different pathways depending on its localisation [5]. There is an increasing number of studies highlighting the expression of PSMA in various solid tumours and growing interest in exploring its potential utilization in different disorders.

The physiological role of this molecule is mostly related to enzymatic peptidase activity related to folate and glutamate metabolism, as well as the activation of signalling pathways [6]. Physiologically, PSMA is expressed in various normal tissues, including prostate epithelium, the small intestine, renal tubules, and salivary glands. In prostate cancer, this protein is overexpressed by 100 to 1000 times, correlating with tumour aggressiveness, which has been successfully utilised in nuclear imaging by employing [^68^Ga]Ga-labelled or [^18^F]F-labelled PSMA as a PET radiotracer for detecting prostate cancer lesions [7].

Already in 1999, Chang et al. proved the presence of PSMA in different neoplastic cell types, such as breast cancer, kidney cancer, bladder cancer, or melanoma [8]. Several studies have demonstrated that PSMA expression is associated with the invasiveness of prostate cancer cells. Its high expression was detected in the neovasculature of most solid tumours, where it regulates angiogenic endothelial cell invasion [6,9,10,11]. Conway et al. indicated that PSMA regulates integrin activation and signal transduction to direct angiogenic endothelial cell adhesion and invasion [12].

However, in clinical practice, PSMA overexpression is used only in prostate cancer. For several years, PSMA ligands have been successfully used to analyse the distribution of prostate cancer cells. As a highly sensitive method, the PET/CT with ^68^Ga-labelled PSMA ligands has become a routine tool for the imaging of prostate cancer, for both staging and detection of recurrence [6,7].

In this manuscript, we report a case of a patient with a parathyroid adenoma visualised only by ^68^Ga-PSMA-11 PET/CT, which indicates a new possible imaging modality for PHPT.

## 2. Results—Case Presentation

A 77-year-old man was hospitalised in the Department of Endocrinology in March 2023 due to primary hyperparathyroidism. The patient was diagnosed with prostate cancer in 2014 and was under regular oncological surveillance. Prior to the hospitalisation, in June 2022, ^68^Ga-PSMA-11 PET/CT was performed to exclude prostate cancer dissemination.

**Figure 1:**
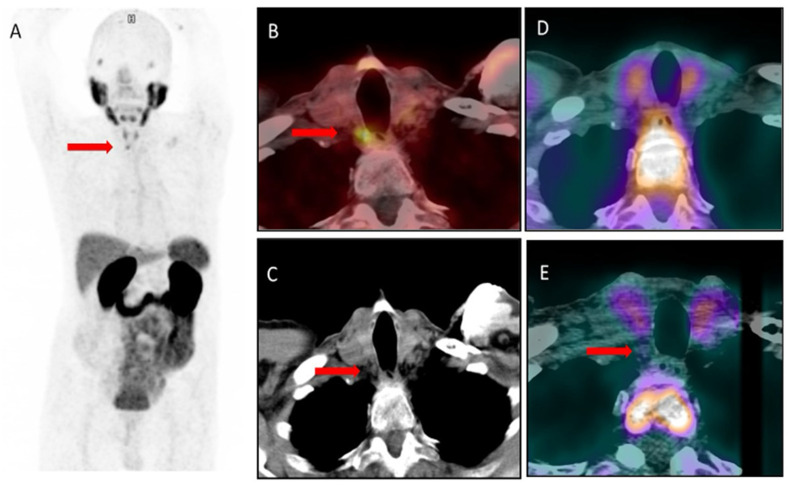
Parathyroid adenoma (red arrows) visualized in ^68^Ga-PMSA-11 PET/CT ((**A**)—PET image, (**B**)—fused transaxial PET/CT image, (**C**)—transaxial CT image). In the ^99m^Tc-MIBI scan ((**D**)—fused transaxial SPECT/CT image) and in ^18^F-fluorocholine PET/CT ((**E**)—fused transaxial PET/CT image), the lesion is visible in the CT, but it shows no uptake of the radiotracer.

Incidentally, the scan indicated increased tracer uptake (SUVmax 6.4) in a lesion below the right thyroid lobe, paravertebrally, measuring 6 × 5 mm (Figure 1A–C). As the focus was not characteristic of prostate cancer metastasis, a suggestion of parathyroid adenoma was raised, and it was confirmed by laboratory evaluation. Consequently, ^99m^Tc-MIBI scintigraphy, including SPECT/CT, was performed. It revealed no increased tracer uptake suggestive of a parathyroid adenoma (Figure 1D). Interestingly, previously, in 2019, due to elevated PSA concentration, the patient had undergone PET/CT using ^18^F-fluorocholine. In a retrospective evaluation of the images, the reported small lesion was present in the CT image, but it did not show any uptake of ^18^F-choline, so it was not reported (Figure 1E).

**Figure 2:**
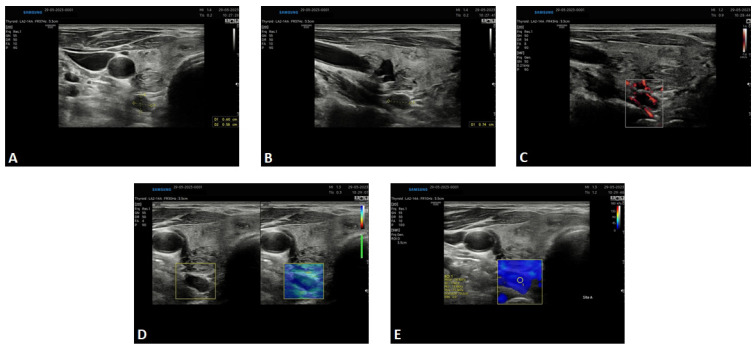
Ultrasound picture of the lesion corresponding to the parathyroid adenoma. The USG was performed March 2023. (**A**)—conventional ultrasound examination; transverse section of the neck; hypoechogenic oval lesion on the postero-lateral wall of the thyroid, located extracapsularly. (**B**)—conventional ultrasound examination; a longitudinal section of the neck; hypoechogenic oval lesion on the posteriori wall of the thyroid, located extracapsularly. (**C**)—the polar vessel sign on superb microvascular imaging. (**D**)—high elasticity of the lesion depicted by strain elastography. (**E**)—high elasticity of the lesion depicted by shear wave elastography. All features suggestive of parathyroid adenoma.

At admission, the patient presented no signs or symptoms of hyperparathyroidism. However, in laboratory assessment, the following features of primary hyperparathyroidism were observed: elevation of total calcium (10.7 mg/dL, normal range up to 10.2 mg/dL) and ionised calcium levels (5.85 mg/dL, normal range up to 5.2 mg/dL) together with hypophosphatemia and increased PTH level (75 pg/mL with a normal range up to 60 pg/mL). In ultrasound, a hypoechogenic lesion was demonstrated, sized 7 × 5 × 9 mm, located behind the lower pole of the right thyroid lobe, consistent with the lesion detected in the ^68^Ga-PSMA-11 PET/CT (Figure 2). Furthermore, the superb microvascular imaging revealed the presence of the polar vessel sign; the strain elastography demonstrated a high level of elasticity in the depicted area, while shear wave elastography also showed a similar high elasticity in the lesion. Parathormone assessment in the washout samples obtained by fine-needle aspiration biopsy from the lesion revealed an extremely high concentration equal to 3264 pg/mL. Moreover, a densitometry examination revealed osteopenia. Abdominal ultrasound demonstrated nephrolithiasis. Based on the whole clinical presentation, primary hyperparathyroidism was diagnosed, and the patient was referred for surgery. The post-surgery histopathological examination confirmed the presence of a parathyroid adenoma.

## 3. Discussion

There are studies that indicate that PET/CT with radiolabelled PSMA ligands could be used as an imaging modality in various malignancies, not only prostate cancer [13]. At our centre, we conducted a clinical study evaluating the potential use of ^18^F-PSMA1007 PET/CT imaging in patients with triple-negative breast cancer (TNBC). This preliminary study showed good performance of ^18^F-PSMA-1007 PET/CT in the detection of TNBC lesions. ^18^F-PSMA-1007 showed high accumulation in distant metastases, higher than in the standard PET/CT imaging using ^18^F-FDG [14].

Nonprostatic diseases exhibiting PSMA uptake on PET are becoming more common as the number of performed scans increases. Few case reports presented incidental findings of parathyroid adenoma in PET/CT with PSMA radiotracers. Subsequently, parathyroidectomy was performed, and a histopathological examination confirmed a parathyroid adenoma [15,16].

The increased uptake of PSMA ligands in the parathyroid adenoma might be related to the tracer’s particular affinity to the focal changes presenting neovascularisation [13]. Pfob et al. and Karimov et al. presented similar cases with incidental findings of parathyroid adenoma in ^68^Ga-PSMA PET/CT examination [12,17]. The post-surgery histopathology revealed that PSMA was mainly overexpressed in small intraparathyrnoidal blood vessels, which is in line with previous reports showing the expression of PSMA in highly vascular regions [18].

Our case and previously published papers suggest that PSMAPET/CT imaging might be a promising new tool for visualising parathyroid adenoma. However, the sensitivity and specificity of this tool in clinical practice need to be evaluated in larger cohort studies.

## 4. Conclusions

This finding suggests that the PSMA ligands’ particular affinity for neovascularisation in focal changes may facilitate the visualisation of parathyroid adenomas. The utilisation of ^68^Ga-PSMA-11 PET/CT in primary hyperparathyroidism could potentially improve the preoperative localization of parathyroid adenomas when conventional imaging methods are inconclusive.

## Data Availability

The data presented in this study are available on request from the corresponding author.
